# A rare case of a familial form of nonsyndromic trigonocephaly

**DOI:** 10.11604/pamj.2015.21.264.6706

**Published:** 2015-08-07

**Authors:** Salahiddine Saghir, Anass Ayad

**Affiliations:** 1 Military Hospital Mohammed V, Rabat, Morocco

**Keywords:** Trigonocephaly, metopic suture, craniosynostosis

## Image in medicine

Isolated trigonocephaly is a nonsyndromic form of craniosynostosis characterized by the premature fusion of the metopic suture. Incidence is estimated at 1/15,000 births. Males are more frequently affected than females (sex ratio of 2:1) and the frequency of trigonocephalic twins is unexpectedly high. The premature closure of the metopic suture results in deformation of the anterior portion of the calvarium and a triangular-shaped forehead. In mild forms, only prominent ridging of the metopic suture is visible; while in more severe forms marked narrowing of the frontal and temporal regions affects the supraorbital rims leading to hypotelorism. The psychomotor development of patients is usually normal and the majority of cases are mild. Most cases are sporadic but familial forms with apparently autosomal dominant transmission have been reported (7-8%). However, the concordance rate of isolated trigonocephaly in monozygotic twins is 43%, suggesting that both genetic and environmental factors are involved in the etiology of this disorder. We report the case of a male newborn infant, having a notion of craniosynostosis in siblings (first brother) and family, the vaginal delivery was at 39 weeks of gestation, his birth weight was 3000 g, length 52 cm, and head circumference 34 cm, the Apgar score was 10/10/10, the clinical examination suspected trigonocephaly (B,C) without other birth defects, the diagnosis was confirmed by a CT scan, the brain parenchyma and the karyotype were normal (A,D). The child was followed in consultation until the age of 1 year and presented normal psychomotor development.

**Figure 1:**
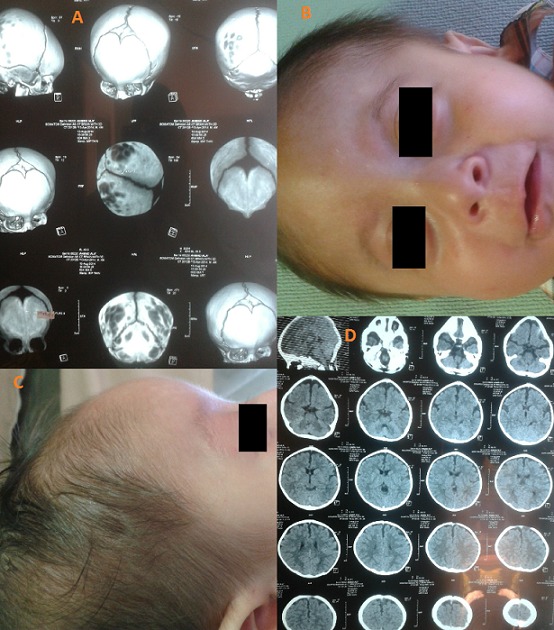
(A) the metopic suture fusion at CT-Scan; (B,C) craniosynostosisat the clinical examination; (D) normal brain parenchyma at CT-Scan

